# Comparison of protocols and RNA carriers for plasma miRNA isolation. Unraveling RNA carrier influence on miRNA isolation

**DOI:** 10.1371/journal.pone.0187005

**Published:** 2017-10-27

**Authors:** Luis A. Ramón-Núñez, Laura Martos, Álvaro Fernández-Pardo, Julia Oto, Pilar Medina, Francisco España, Silvia Navarro

**Affiliations:** Grupo de Hemostasia, Trombosis, Arteriosclerosis y Biología Vascular, Instituto de Investigación Sanitaria La Fe (IIS La Fe). Hospital Universitario y Politécnico La Fe, Valencia, Spain; Oxford Brookes University, UNITED KINGDOM

## Abstract

microRNAs are promising biomarkers in biological fluids in several diseases. Different plasma RNA isolation protocols and carriers are available, but their efficiencies have been scarcely compared. Plasma microRNAs were isolated using a phenol and column-based procedure and a column-based procedure, in the presence or absence of two RNA carriers (yeast RNA and MS2 RNA). We evaluated the presence of PCR inhibitors and the relative abundance of certain microRNAs by qRT-PCR. Furthermore, we analyzed the association between different isolation protocols, the relative abundance of the miRNAs in the sample, the GC content and the free energy of microRNAs. In all microRNAs analyzed, the addition of yeast RNA as a carrier in the different isolation protocols used gave lower raw Cq values, indicating higher microRNA recovery. Moreover, this increase in microRNAs recovery was dependent on their own relative abundance in the sample, their GC content and the free-energy of their own most stable secondary structure. Furthermore, the normalization of microRNA levels by an endogenous microRNA is more reliable than the normalization by plasma volume, as it reduced the difference in microRNA fold abundance between the different isolation protocols evaluated. Our thorough study indicates that a standardization of pre- and analytical conditions is necessary to obtain reproducible inter-laboratory results in plasma microRNA studies.

## Introduction

microRNAs (miRNAs) are non-coding RNAs of 16–28 nucleotides, that regulate gene expression and play fundamental roles in biological processes such as differentiation, development, cell signalling and response to infections [1]. These small molecules bind to mRNA targets and function as post-transcriptional gene regulators, through translational repression and/or mRNA degradation [1,2]. Each miRNA is able to target hundreds of mRNAs, and different miRNAs are able to target the same mRNA. miRNAs have a tissue and cell type-dependent expression pattern [3,4] and abnormal miRNA expression is associated with several human diseases, including cancer [5–7], cardiovascular [8,9], gynecological [10], inflammatory and autoimmune diseases [11,12]. As miRNAs are very stable and easy to detect in different biological fluids such as plasma, urine or saliva [13–15], they have been postulated as ideal non-invasive diagnostic and prognostic biomarkers. This stability is due to the fact that miRNAs circulate in plasma within exosomes, microparticles and apoptotic bodies [16] or protected by Argonaute 2 complexes [17,18].

Different studies have been previously conducted to compare the efficiency of RNA isolation using different miRNA isolation protocols in cell supernatant medium, serum or plasma samples [19–25]. These comparisons were mainly made by quantitative reverse transcriptase polymerase chain reaction (qRT-PCR) [21–25], the gold-standard for RNA quantification. However, only a few studies have employed mainly spectrometry or electropherogram analysis without qRT-PCR [19,20] to conduct these comparisons. Furthermore, little attention has been paid to the evaluation of possible qRT-PCR inhibitors co-purified during the RNA isolation protocols.

It is well known that the addition of a RNA carrier during the RNA isolation procedure increases miRNA recovery, and therefore the subsequent miRNA detection [26]. However, we must consider that this RNA carrier may mask the final yield of isolated RNA by modifying the spectrophotometric quantification and the electropherogram analyses. RNA carriers also affect the quality of the analysis, by increasing the A260/230 ratio, and could cause changes in the miRNA profile obtained from plasma [20]. Only a few studies have been conducted to address the effectiveness of different RNA carriers in RNA isolation [25,27]. Finally, it has been described that structured small RNAs with low GC content (miRNAs, premiRNAs and tRNAs) seem to be inefficiently recovered when a small number of cells are used in RNA isolation using phenol-based isolation procedures, but this effect has not been observed in column-based protocols [28]. Consequently, care should be taken with samples with low small–RNA content, such as serum, plasma or cell culture medium, because certain miRNAs may be selectively lost during RNA isolation depending on the procedure of choice [28].

As phenol-based procedures seem to result in poor recovery of miRNAs with low GC content from samples with low RNA abundance [28], we quantified by qRT-PCR different miRNAs (hsa-miR-15a-5p, hsa-miR-16-5p, hsa-miR-21-5p, hsa-miR-23a-3p, hsa-miR-23b-3p, hsa-miR-24-3p, hsa-miR-25-3p, hsa-miR-30d-5p, hsa-miR-93-5p, hsa-miR-101-3p, hsa-miR-103a-3p, hsa-miR-106b-5p, hsa-miR-122-5p, hsa-miR-126-3p, hsa-miR-144-3p, hsa-miR-185-5p, hsa-miR-223-3p, hsa-miR-320a, hsa-miR-451a, hsa-let-7a-5p, hsa-let-7b-5p, and hsa-let-7g-5p) with different GC content and thermodynamic stability in several RNA samples isolated from plasma using different protocols and RNA carriers. These analyses allowed us to detect the bias in miRNA composition depending on the RNA isolation protocol used.

This is one of the few studies in which miRNA isolation efficiency is compared using different RNA isolation protocols in combination with different RNA carriers [25,27]. For this purpose, we have tested by qRT-PCR the presence of PCR inhibitors, the relative abundance of the aforementioned miRNAs with different GC content and thermodynamic stability in plasma-derived RNA samples obtained using two different protocols in the presence or absence of two different RNA carriers. Furthermore, we also compared two different normalization strategies in order to acknowledge the strategy that renders the lowest variability between isolation protocols.

## Materials and methods

### Ethics statement

The research was carried out according to The Code of Ethics of the World Medical Association (Declaration of Helsinki). Written informed consent was obtained from all individuals, and the study was approved by the Ethical Committee from Hospital Universitario y Politécnico La Fe, Valencia, Spain (#2012/0149).

### Blood collection and sample processing

Blood samples were obtained from ten healthy subjects. Platelet poor plasma was obtained by centrifugation at 1500 x g for 30 minutes at 4°C. Collection of platelet poor plasma was stopped 1 cm above the buffy coat to avoid cell contamination. The platelet poor plasma was stored in aliquots at -80°C until further analysis.

### RNA isolation

The quality and isolation efficiency of miRNAs were analyzed in four plasma samples using two protocols: phenol and column based isolation procedures (miRNeasy Mini kit, Qiagen, Q protocol) and column based procedures (miRCURY RNA Isolation kit Biofluids, Exiqon, E protocol).

The isolation protocols were performed according to manufacturer’s instructions with the following modifications: Plasma samples were thawed in melting ice and centrifuged at 3,000 x g at 4°C for 5 min to avoid the presence of cell debris in the sample. A volume of 200 μl of plasma was transferred to a new microcentrifuge tube as starting material in all the protocols. Then, 1 ml of QIAzol Lysis Reagent for the Q protocol or 60 μl of Lysis Solution BF for the E protocol was added and the mixture was incubated for 5 min at room temperature. Next, 1 μg of RNA carrier (Torulla Ambion yeast RNA, Life Technologies; MS2 bacteriophage RNA, Roche; or no carrier) and 1 μl of synthetic miRNA mix (UniSp2, UniSp4, and UniSp5 from RNA Spike-in kit UniRT, Exiqon) were added in both protocols. Optimum RNA carrier concentration was empirically determined (S1 Fig). Finally, an additional washing step with RPE Buffer was performed in the Q protocol. In addition, we performed the same isolation protocols using 200 μl of water as starting material to obtain the no-template control sample, in order to detect downstream unspecific amplifications. Isolated RNA was stored at -80°C.

After the analysis of preliminary results, total RNA from sixteen extra plasma control samples was isolated with Q and E protocols in presence or absence of yeast RNA carrier in order to check the isolation efficiency of small RNAs with different GC content and thermodynamic stability. Thermodynamic stability was evaluated by the free energy of the most stable secondary structure of miRNAs (miRNA ΔG).

### RNA concentration, quality, and integrity

The RNA concentration and quality were determined using a NanoDrop ND-1000 spectrophotometer (Thermo Fisher Scientific). We used the A260/280 and A260/230 ratios to check for possible co-purified contaminants during the RNA isolation (S1 Table).

RNA concentration and integrity were also analyzed using the Agilent RNA 6000 Nano Kit for total RNA and Agilent Small RNA kit for low molecular weight RNA in the Agilent 2100 Bioanalyzer (Agilent Technologies) (S2 Table and S2 Fig).

### qRT-PCR

2 μl of RNA were reverse transcribed in duplicate in a final reaction volume of 10 μl using the Universal cDNA Synthesis kit II (Exiqon). This input RNA volume was empirically adjusted (S1 Fig). The Master Mix contained 2 μl of 5X Reaction Buffer, 0.5 μl of synthetic UniSp6 RNA spike-in template resuspended according to the manufacturer instructions, 1 μl of 10X Enzyme Mix and 4.5 μl of nuclease-free water. The reactions were performed in a TC-412 Thermocycler (Techne) for 60 min at 42°C, 5 min at 95°C, and then were cooled to 4°C and stored to -80°C until used.

In the qPCR step, each 10 μl reaction contained 4 μl of cDNA sample (previously diluted 1/40), 5 μl of ExiLENT SYBR® Green master mix (Exiqon) and 1 μl of microRNA LNA™ PCR primer mix (Exiqon). qPCRs were performed following manufacturer's instructions using the LightCycler 480 real-time PCR system (Roche) in 384 well plates, and each sample was amplified in duplicate. After denaturing the cDNA and activating the enzyme polymerase for 10 min at 95°C, the cycling conditions were as follows: 45 cycles consisting of denaturation at 95°C for 10 s and then annealing/extension at 60°C for 60 s (with ramp rate of 1.6°C/s). Cq values, using the second derivative method, and melting curves were obtained using the LightCycler 480 software (Roche).

### Evaluation of the isolation efficiency and PCR inhibition using synthetic UniSp2 RNA spike-in

We used serial tenfold dilutions of known concentrations of the synthetic UniSp2 RNA Spike-in (UniSp2) to create a standard curve. The slope of the plot was used to calculate the qPCR efficiency (1).

$$qPCR\;EFF(\%)=\left(10^{\left(-1/slope\right)}-1\right)\;x\;100$$ (1)

It is unknown whether differences in Cq values between isolation protocols arise from different RNA recovery efficiencies, different inhibitor removal efficiencies, or both. Therefore, we evaluated the qPCR Eff in four samples isolated with the Q and E protocols in the presence or absence of RNA carrier (yeast RNA or MS2 bacteriophage RNA), performing a tenfold serial dilutions experiments of each sample and quantifying the UniSp2. The results of the slope of each sample were also translated into a value for qPCR Eff. When no differences are observed between standard and sample qPCR Eff values, we can assume that qPCR is not influenced by inhibitors, thus differences observed between isolation protocols are caused by different isolation efficiencies (Isol. Eff).

The concentration of each sample was calculated by simple interpolation of its Cq into the standard curve, and the Isol. Eff was calculated (2) using the following parameters: “UniSp2 CC” the absolute concentration of UniSp2 recovered calculated by qRT-PCR, and “UniSp2 TMC” the theoretical maximum concentration of UniSp2 that can be recovered.

$$Isol\;Eff(\%)=\left(\frac{UniSp2\;CC}{UniSp2\;\mathrm{TMC}}\right)\;x\;100$$ (2)

### Evaluation of Cq values between protocols and RNA carriers

**Fig 1:**
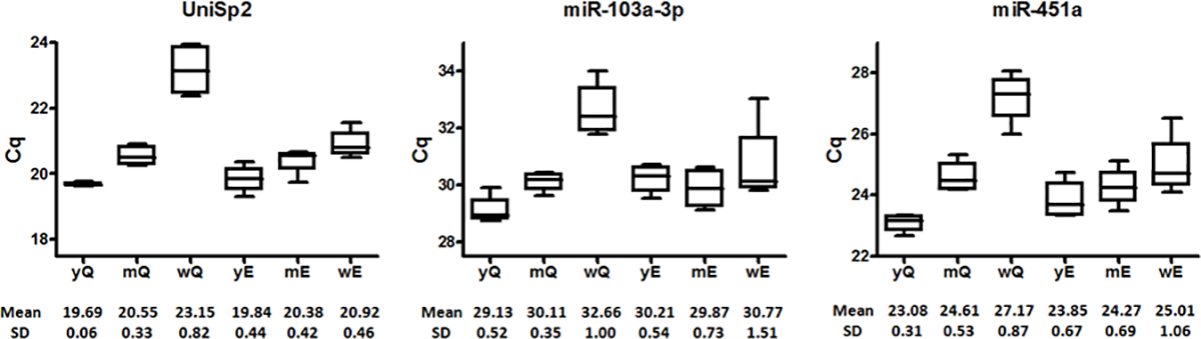
Raw Cq values. Evaluation of Cq values of UniSp2, miR-103a-3p and miR-451a in four samples using different isolation protocols and RNA carriers. y, yeast RNA carrier; m, MS2 RNA carrier; w, without carrier; Q, miRNeasy Mini kit modified protocol; E, miRCURY RNA isolation kit Biofluids modified protocol. Mean and standard deviation values are indicated under each box plot diagram.

In an initial approach, we evaluated Cq values of the synthetic UniSp2 and UniSp6 RNA spike-in (Exiqon) and the miRNAs hsa-miR-103a-3p and hsa-miR-451a in four plasma-derived RNA samples, isolated with Q and E modified protocols, in presence of yeast RNA (yQ and yE) or MS2 bacteriophage RNA (mQ and mE) as carrier and also without RNA carrier (wQ and wE) (Fig 1). We used hsa-miR-103a-3p, a well-known tissue and plasma endogenous normalizer [29,30] and hsa-miR-451a, a marker of hemolysis [31]. After the analysis of preliminary results we studied more miRNAs (see next section) and compared the Cq values obtained with the different RNA isolation protocols used.

### Evaluation of the fold recovery of miRNAs with different GC content and ΔG

We evaluated the “fold recovery” (FR) of several miRNAs with different GC content and intra-molecular ΔG in twenty plasma-derived RNA samples isolated with the Q and E modified protocols in presence (y) or absence (w) of yeast RNA carrier (yQ, yE, wQ, and wE). We defined FR as the miRNA mean abundance normalizing by plasma volume and taking as reference the protocols without RNA carrier (i.e., wQ or wE protocols). It shows us how much miRNA is recovered after carrier addition when it is referred to the same protocol without carrier, for instance, yE referred to wE.

Table 1 shows the main characteristics of the different miRNAs analyzed: sequence (according to miRBase 21.0), nucleotide number, GC content (%), miRNA ΔG (folding free energy predicted by Mfold [32]).and sample relative abundance (SRA) of each miRNA, i.e. the mean miRNA abundance taking as reference the mean Cq value of all miRNAs used in the wQ or wE protocols. Relative quantification was calculated using plasma volume normalization (S5 Table) and the mean Cq (3), being the Cq average of all miRNAs analyzed from all samples and protocols used [33].

$$Relative\;Quantification=2^{\left(mean\;Cq-Cq\;test\;sample\right)}$$ (3)

**Table 1:** Main characteristics of the miRNAs analyzed by qRT-PCR.

miRNA	Sequence (5’–3’)	Length (number of nucleotides)	%GC	miRNA ΔG (Kcal/mol)	miRNASRA (wQ)	miRNASRA (wE)
**UniSp2**	**x**	**x**	**47.62**	**-2.3**	**20.639**	**20.736**
**hsa-let-7a-5p**	**UGAGGUAGUAGGUUGUAUAGUU**	**22**	**36.36**	**2.1**	**0.023**	**0.009**
**hsa-let-7b-5p**	**UGAGGUAGUAGGUUGUGUGGUU**	**22**	**45.45**	**3.3**	**0.013**	**0.014**
**hsa-let-7g-5p**	**UGAGGUAGUAGUUUGUACAGUU**	**22**	**36.36**	**0.0**	**0.016**	**0.013**
**hsa-miR-15a-5p**	**UAGCAGCACAUAAUGGUUUGUG**	**22**	**40.91**	**-2.1**	**0.047**	**0.039**
**hsa-miR-16-5p**	**UAGCAGCACGUAAAUAUUGGCG**	**22**	**45.45**	**-1.4**	**0.701**	**0.795**
**hsa-miR-21-5p**	**UAGCUUAUCAGACUGAUGUUGA**	**22**	**36.36**	**-1.0**	**0.133**	**0.072**
**hsa-miR-23a-3p**	**AUCACAUUGCCAGGGAUUUCC**	**21**	**47.62**	**-1.0**	**0.106**	**0.124**
**hsa-miR-23b-3p**	**AUCACAUUGCCAGGGAUUACC**	**21**	**47.62**	**-0.3**	**0.045**	**0.051**
**hsa-miR-24-3p**	**UGGCUCAGUUCAGCAGGAACAG**	**22**	**54.55**	**-3.2**	**0.086**	**0.089**
**hsa-miR-25-3p**	**CAUUGCACUUGUCUCGGUCUGA**	**22**	**50.00**	**1.1**	**0.046**	**0.036**
**hsa-miR-30d-5p**	**UGUAAACAUCCCCGACUGGAAG**	**22**	**50.00**	**-1.7**	**0.021**	**0.019**
**hsa-miR-93-5p**	**CAAAGUGCUGUUCGUGCAGGUAG**	**23**	**52.17**	**-3.4**	**0.017**	**0.018**
**hsa-miR-101-3p**	**UACAGUACUGUGAUAACUGAA**	**21**	**33.33**	**-2.1**	**0.011**	**0.006**
**hsa-miR-103a-3p**	**AGCAGCAUUGUACAGGGCUAUGA**	**23**	**47.83**	**-1.1**	**0.029**	**0.021**
**hsa-miR-106b-5p**	**UAAAGUGCUGACAGUGCAGAU**	**21**	**42.86**	**-2.9**	**0.009**	**0.007**
**hsa-miR-122-5p**	**UGGAGUGUGACAAUGGUGUUUG**	**22**	**45.45**	**-0.1**	**0.066**	**0.025**
**hsa-miR-126-3p**	**UCGUACCGUGAGUAAUAAUGCG**	**22**	**45.45**	**-0.7**	**0.081**	**0.050**
**hsa-miR-144-3p**	**UACAGUAUAGAUGAUGUACU**	**20**	**30.00**	**-3.3**	**0.009**	**0.008**
**hsa-miR-185-5p**	**UGGAGAGAAAGGCAGUUCCUGA**	**22**	**50.00**	**-1.3**	**0.047**	**0.026**
**hsa-miR-223-3p**	**UGUCAGUUUGUCAAAUACCCCA**	**22**	**40.91**	**0.7**	**0.575**	**0.595**
**hsa-miR-320a**	**AAAAGCUGGGUUGAGAGGGCGA**	**22**	**54.55**	**-1.2**	**0.040**	**0.016**
**hsa-miR-451a**	**AAACCGUUACCAUUACUGAGUU**	**22**	**36.36**	**1.4**	**1.210**	**1.213**
**hsa-miR-486-5p**	**UCCUGUACUGAGCUGCCCCGAG**	**22**	**63.64**	**0.9**	**0.029**	**0.018**

miRNA SRA (wQ), miRNA relative abundance in the sample taking as reference the wQ isolation protocol; miRNA SRA (wE), miRNA relative abundance in the sample taking as reference the wE isolation protocol; X, no information provided by manufacturer.

This type of normalization can theoretically be performed because identical sample and elution volumes were used in each isolation protocol. The FR for each isolation protocol was calculated using as reference the average of results of the relative quantification using wE or wQ protocols (S5 Table). Finally, correlations between FR values, miRNA SRA, GC content, and miRNA ΔG of each miRNA were calculated.

### Evaluation of different normalization strategies

We evaluated two normalization strategies in order to acknowledge the most consistent. Thus, we compared differences in “fold abundance” values between different protocols within the same normalization strategy. The normalization strategies were: a) plasma volume normalization (S5 Table) and b) normalization using an endogenous miRNA (hsa-miR-103a-3p) [29,30] (S6 Table). Relative quantification normalized by plasma volume was done as described before [33], and relative quantification normalized by hsa-miR-103a-3p was done using the 2^-ΔΔCt^ method [34]. In both cases we took as reference the mean value for wE or wQ protocols (S5 and S6 Tables). We must emphasise that “fold abundance” values obtained normalizing by plasma volume were “fold recovery” (FR) of miRNAs, i.e., how much quantity of a miRNA is recovered by a protocol with regard a reference protocol. Using without carrier protocols (wE or wQ) as reference protocols we are able to detect how much miRNA is recovered after adding the RNA carrier. However, normalizing by an endogenous miRNA we are not able to obtain the FR precisely because this endogenous miRNA is recovered with a similar efficiency that the miRNA target. Furthermore, the recovery efficiency of a given miRNA vary between different isolation protocols. In this case, the “fold abundance” values obtained normalizing by an endogenous miRNA were “apparent fold change” (apFC) values. We are able to speak of apFC because data were obtained of the same twenty samples. Thus, no real differences were between them. For example, an increase in the “fold abundance” of data normalized by miR-103a-3p, will be due to a higher recovery of target miRNA or to a lower recovery of miR-103a-3p regarding the reference protocol. Therefore, we will observe an increase in the apFC for these specific miRNA.

### Statistical analysis

Comparisons of RNA concentration, raw Cq values, miRNA values normalized by plasma volume or by miRNA-103a-3p, were performed using the two-tailed Wilcoxon Signed Ranks Test with the IBM SPSS Statistics 20 software. Correlations were calculated using the two-tailed bivariate Pearson correlation test. Stepwise multiple regression analyses were used to calculate the variables that predict variance in FR. The predictor variables included the miRNA SRA, base 10 logarithm of miRNA SRA, the GC content, and the miRNA ΔG. *P*-values <0.05 were considered significant.

## Results

In this work we compared different modified isolation protocols (miRCURY RNA isolation kit Biofluids [Exiqon] and, miRNeasy Mini protocol [Qiagen]) named here as E and Q protocols. Furthermore, these isolation protocols were used with two different types of RNA carrier (Torulla yeast RNA carrier and MS2 bacteriophage RNA carrier) and without RNA carrier. After preliminary results we discarded MS2 carrier.

### RNA concentration, quality and integrity

The RNA concentrations estimated by spectrometry, determined as a peak at 260 nm, were different between each isolation protocol and RNA carrier employed. The mean values ranged from 9.4 ng/μl obtained by the wQ protocol, to 27.7 ng/μl obtained by the mQ protocol (S1 Table). Similar results were obtained between Q and E modified protocols when yeast RNA or MS2 RNA were used as carriers. In the absence of RNA carriers, the E modified protocol gave the highest RNA recovery. Differences were not statistically significant.

The A260/280 and A260/230 ratios were used to assess the purity of RNA (S1 Table). Ratios of ~2.0 are generally accepted as high quality for RNA. Low A260/280 and A260/230 ratios indicate the presence of protein, phenols or other carryover contaminants that absorb strongly at or near 280 and 230 nm. Thus, low quality RNA was obtained with all combinations of different protocols and carriers. The mQ protocol was the only one that showed a relatively good A260/280 coefficient.

Total RNA concentrations obtained with the Agilent 2100 Bioanalyzer were lower than those obtained by spectrometry (S2 Table and S2 Fig). The highest total RNA concentration was observed using the mQ protocol, although electropherogram analyses showed a smear signal over all RNA regions. As yeast RNA carrier has a size range similar to small RNAs, we could observe the highest small RNA and miRNA concentrations using this RNA carrier in both Q and E modified protocols, and the electropherogram analysis also showed a peak in the miRNAs region (S2 Fig). On the other hand, the electropherogram analysis also showed that the E protocol yielded the lowest small RNA and miRNA concentrations (S2 Table).

### Evaluation of the isolation efficiency and PCR inhibition using synthetic UniSp2 RNA Spike-in

Similar qPCR Eff was observed with the standard curve (87%) and the ten-fold diluted samples (85–91%) (S3 Table), indicating that there was no qPCR inhibition. Therefore, differences in Cq values observed between different protocols were due to distinct isolation efficiencies for the different protocols used and were not due to the presence of inhibitors that theoretically would alter the PCR reaction. In addition, the highest UniSp2 spike-in recovery and, therefore, isolation efficiency, was observed when RNA carrier was used. Furthermore, we observed an increased UniSp2 recovery with both protocols when yeast RNA was used as RNA carrier (S3 Table). In the absence of carrier, we observed a higher UniSp2 concentration, and therefore a higher recovery, with the wE than with the wQ protocol (S3 Table).

### Evaluation of Cq values between protocols and RNA carriers

The highest miRNA concentration (lowest raw Cq values) was obtained using the yQ protocol in all miRNAs studied compared to the other isolation protocols used (Fig 1 and S4 Table), whereas the lowest miRNA concentration (highest raw Cq values) was obtained in the absence of RNA carrier in all cases (Fig 1 and S4 Table). As expected, no differences were observed in the Cq values of UniSp6 spike-in between different isolation protocols (S4 Table). Thus, no PCR inhibition was observed using the different isolation protocols. The no-template control sample with carrier (obtained using the same isolation procedure using water instead of serum as starting material) and the RT- control sample did not show signal or Cq values were at least 10 cycles higher than those of the test samples for all miRNAs tested (data not shown). Therefore, we can discard non-specific amplifications in presence of carrier.

### Evaluation of the fold recovery of miRNAs with different GC content and ΔG

When we analyzed the FR values of miRNAs normalized by plasma volume, we observed that the yQ protocol in almost all miRNAs analyzed gave significantly higher FR compared to the other protocols (S5 Table). yE protocol showed higher FR values for all miRNAs tested compared to wQ and wE protocols. Furthermore, wE protocol also showed higher FR than wQ protocol, except for several miRNAs (let-7a-5p, miR-122-5p, and miR-320a), which showed the same FR in wQ protocol. Finally, no differences were observed in UniSp6 spike-in levels between the different isolation protocols (S4 Table).

**Fig 2:**
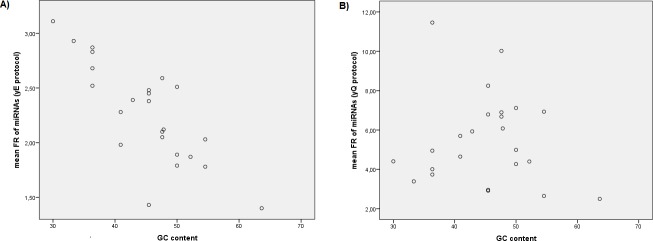
**Correlations between mean “fold recovery” (FR) values of miRNAs and GC content of each miRNA, using yE protocol (A) and yQ protocol (B).** yE, miRCURY RNA isolation kit Biofluids modified protocol using yeast RNA carrier; yQ, miRNeasy Mini kit modified protocol using yeast RNA carrier; FR, the fold recovery of each miRNA vs the same protocol without carrier.

**Fig 3:**
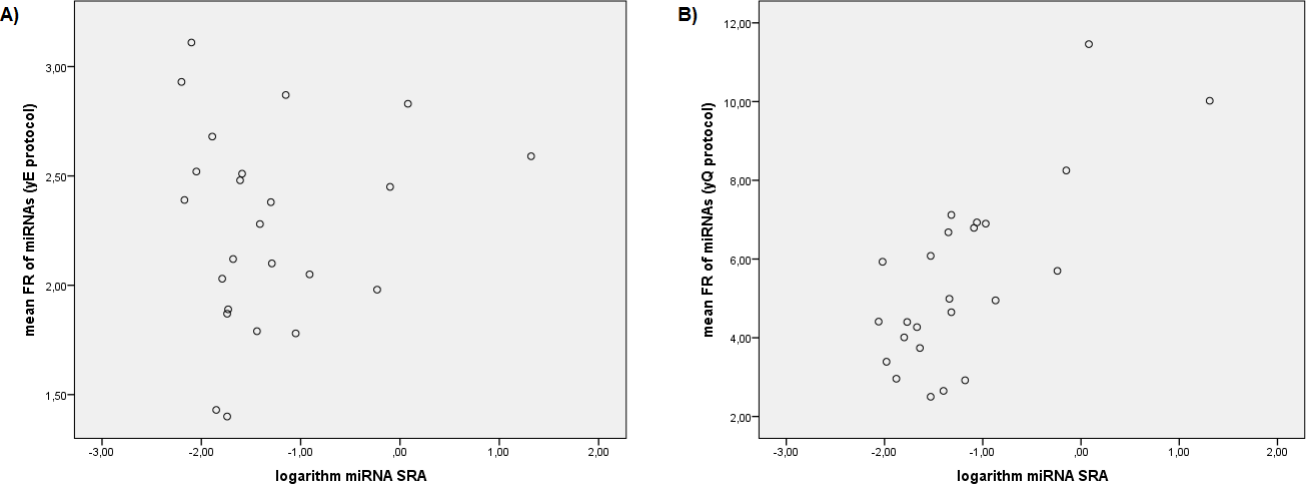
**Correlations between mean “fold recovery” (FR) values of miRNAs and base 10 logarithm of miRNA relative abundance in sample (SRA), using yE protocol (A) and yQ protocol (B).** yE, miRCURY RNA isolation kit Biofluids modified protocol using yeast RNA carrier; yQ, miRNeasy Mini kit modified protocol using yeast RNA carrier; FR, the fold recovery of each miRNA vs the same protocol without carrier; SRA, the mean relative abundance of each miRNA on samples isolated without carrier.

**Fig 4:**
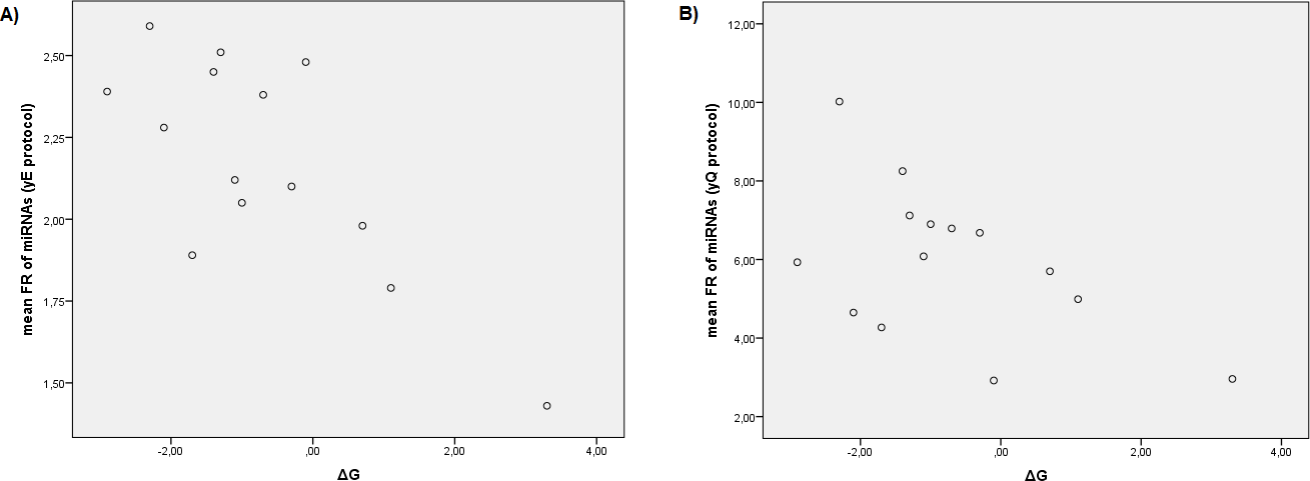
**Correlations between mean “fold recovery” (FR) values of miRNAs and ΔG of each miRNA, using yE protocol (A) and yQ protocol (B).** To avoid the influence of GC content in the analysis of ΔG, for these correlations we used only miRNAs with a GC content between 25% and 75% percentile. yE, miRCURY RNA isolation kit Biofluids modified protocol using yeast RNA carrier; yQ, miRNeasy Mini kit modified protocol using yeast RNA carrier; FR, the fold recovery of each miRNA vs the same protocol without carrier; ΔG, the free energy of the most stable secondary structure of each miRNA.

Significant negative correlations between the individual FR values of miRNAs in yE protocol with the GC content (r = -0.290, *P*<0.001) and the miRNA ΔG (r = -0.103, *P* = 0.023) were observed, but no correlation with miRNA SRA or with the logarithm of miRNA SRA were observed. Furthermore, using mean FR values of miRNAs, the correlation with the GC content improved (r = -0.799, *P*<0.001) (Fig 2A). However, we did not observe correlation with miRNA ΔG, miRNA SRA or the logarithm of miRNA SRA (Fig 3A). On the other hand, a stepwise multiple linear regression analysis was performed using miRNA SRA, the logarithm of miRNA SRA, the GC content and the miRNA ΔG as independent variables and mean FR values of all miRNAs tested for yE protocol as dependent variables. This analysis showed that GC content and miRNA ΔG were predictors of FR for yE protocol and the predictive equation obtained for the FR of miRNAs using yE protocol was: yE = 4.339–0.047 x GC content– 0.078 x miRNA ΔG (adjusted R^2^ = 0.703). To reduce the influence of GC content in the miRNAs analyzed (from 30% to 63%, Table 1) we performed correlations using only miRNAs between percentile 25% and 75%, i.e., with GC content from 38.64% to 50% GC. In this case, significant correlations between the individual FR values of miRNAs in yE protocol with the logarithm of miRNA SRA (r = 0.133, *P* = 0.026), and miRNA ΔG (r = -0.223, *P*<0.001) were observed. When mean FR values for each miRNA were used, the correlation with miRNA SRA was not significant and the correlation with miRNA ΔG increased (r = -0.752, *P* = 0.002) (Fig 4A). This means that the use of yeast RNA carrier in yE protocol increases the recovery efficiency of miRNAs mainly by their own GC content, but also by miRNA ΔG (that is, their own miRNA secondary structure) and in a lesser extent by SRA.

Significant positive correlations between the individual FR values of miRNAs in yQ protocol with the miRNA SRA (r = 0.293, *P*<0.001) and the logarithm of miRNA SRA (r = 0.467, *P*<0.001) were observed. These correlations increased when the mean FR values were used (r = 0.468, *P* = 0.021 and r = 0.745, *P*<0.001) (Fig 3B). However, we did not observe correlations between individual or mean FR values of miRNAs using yQ protocol with GC content (Fig 2B) or with ΔG. Otherwise, a stepwise multiple linear regression analysis was performed using miRNA SRA, the logarithm of miRNA SRA, the GC content and the miRNA ΔG as independent variables (as before) and mean FR values of all miRNAs tested for yQ protocol as dependent variables. In this case, the logarithm of miRNA SRA was a good predictor of FR for yQ protocol and the predictive equation obtained for the FR of miRNAs using yQ protocol was: yQ = 8.086 + 2.167 x logarithm of the relative abundance of miRNAs in the sample (adjusted R^2^ = 0.535). To verify if other variables influence the recovery of the miRNAs and to avoid the influence of very abundant miRNAs in the sample, we removed from the analysis values for UniSp2 spike-in and miR-451a, both overrepresented in the samples, with three and two orders of magnitude above the miRNAs less represented in the sample (miR-106b-5p and miR-144-3p, Table 1). In this case, significant correlations between the individual FR values of miRNAs in yQ protocol with miRNA SRA (r = 0.262, *P*<0.001), the logarithm of miRNA SRA (r = 0.301, *P*<0.001), and miRNA ΔG (r = -0.179, *P*<0.001) were observed. When mean FR values of miRNAs were used, positive correlations with the miRNA SRA and the logarithm of miRNA SRA were observed (r = 0.472, *P* = 0.027, and r = 0.543, *P* = 0.009, respectively). The stepwise multiple linear regression analysis without UniSp2 and miR-451a was repeated using mean FR values as dependent variables. In this case, the logarithm of miRNA SRA and the miRNA ΔG were predictors of FR for yQ protocol and the predictive equation obtained was: yQ = 7.294 + 1.892 x logarithm of miRNA SRA—0.365 x miRNA ΔG (adjusted R^2^ = 0.382). Furthermore, performing correlations using only miRNAs with GC content between 25% and 75% percentile as before, significant correlations between the individual FR values of miRNAs in yQ protocol with the miRNA SRA (r = 0.358, *P*<0.001), the logarithm of miRNA SRA (r = 0.413, *P*<0.001), and miRNA ΔG (r = -0.305, *P*<0.001) were observed. When mean FR values of miRNA were used, significant correlations with miRNA SRA and with the logarithm of miRNA SRA were observed (r = 0.620, *P =* 0.019, and r = 0.710, *P*<0.004, respectively), but the correlation with miRNA ΔG was no significant (r = -0.525, *P* = 0.054) (Fig 4B). This means that the use of yeast RNA carrier in yQ protocol increases the recovery efficiency of miRNAs mainly by their own SRA. That is, the recovery of a determined miRNA depends first and foremost of its own abundance in the sample. But also are important variables, in a lesser degree, the miRNA ΔG and GC content.

Our data showed that the use of RNA carrier increases the isolation efficiency of miRNAs in a specific way, and this recovery depends, to a greater or lesser extent, of miRNA SRA, ΔG and GC content. Furthermore, this isolation efficiency varies between protocols because miRNA SRA, ΔG and GC content have different weight in each protocol. It is important to highlight that these predictive models obtained are dependent of the miRNAs used. In this work we used miRNAs with a difference of about three orders of magnitude of sample relative abundance (SRA), ΔG between -3.4 and 3.3 Kcal/mol and, GC content between 30 to 64%. However, there are miRNAs with more extreme values, for instance, GC content of human mature miRNAs ranges from 8.70 (hsa-miR-2054) to 95.24% (hsa-miR-1908-3p). These data were calculated from human mature miRNAs sequences available on miRBase 21.0 (www.miRbase.org). Thereby, for instance, using miRNAs with extreme GC content could give rise to models in which ΔG, or relative abundance of miRNAs, or both are less important or negligible.

### Evaluation of different normalization strategies

**Fig 5:**
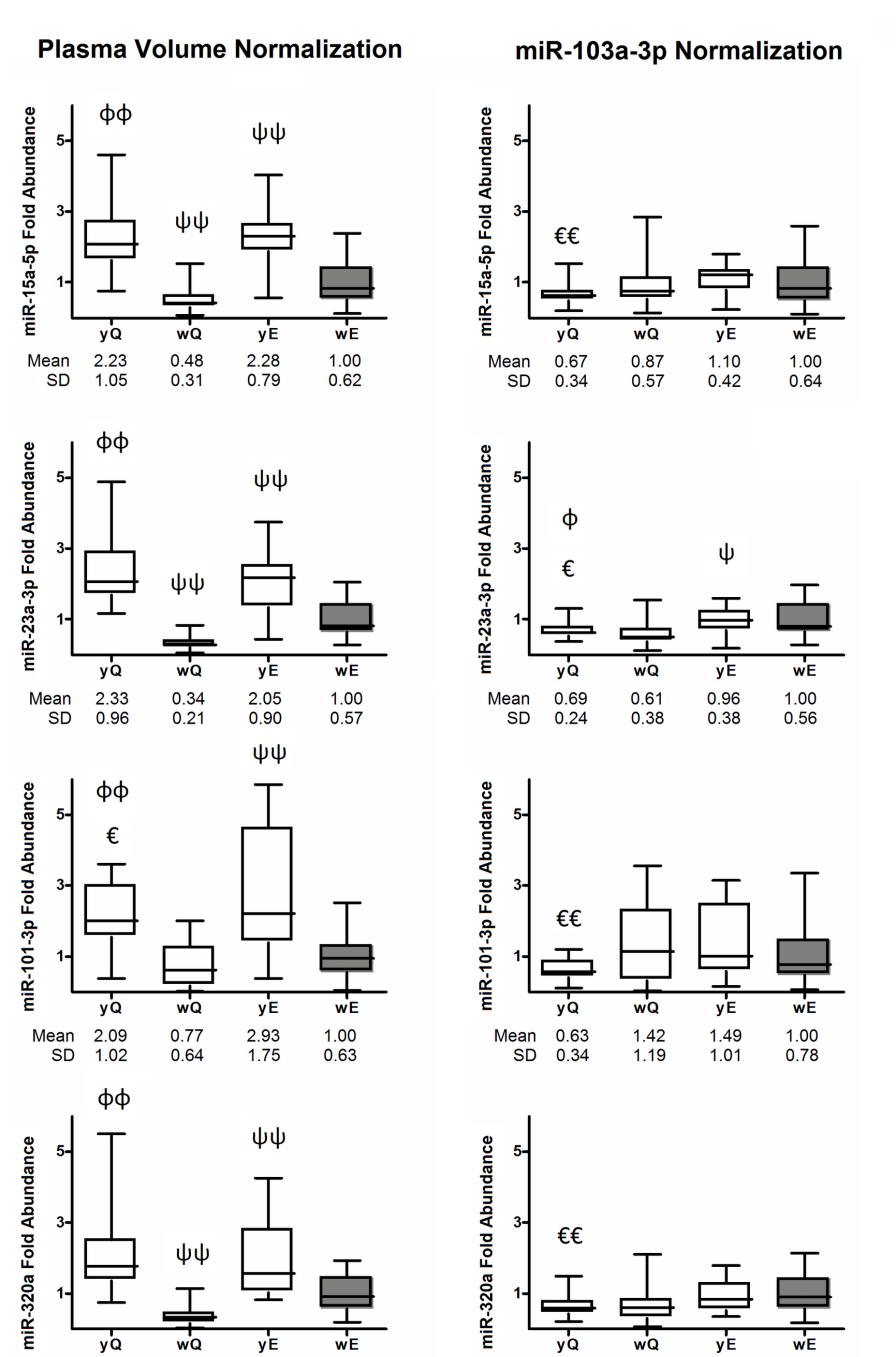
miRNA levels normalized by plasma volume and by an endogenous miRNA. Fold abundance of miRNAs normalized by plasma volume (FR of miRNAs, left column) and normalized by hsa-miR-103a-3p (apFC of miRNAs, right column) and referred to control plasma samples isolated with the E protocol without carrier (in gray). Data were determined in plasma RNA-derived samples isolated with Q and E modified protocols with yeast RNA as carrier (y) or without RNA carrier (w). E, miRCURY RNA isolation kit Biofluids modified protocol; Q, miRNeasy Mini kit modified protocol; FR, fold recovery of miRNAs vs wE protocol; apFC, apparent fold change of miRNAs vs wE protocol. *P*-values were calculated using Wilcoxon Signed Rank Test, Exact Signification two-tailed, with the IBM SPSS Statistics 20 software. ^ϕ^*P*<0.05, and ^ϕϕ^*P*<0.001 vs wQ; ^€^*P*<0.05, and ^€€^*P*<0.001 vs yE; ^ψ^*P*<0.05, and ^ψψ^*P* = 0.001 vs wE. Mean and standard deviation values are indicated under each box plot diagram.

Once we analyzed the “fold abundance” of miRNAs normalized by plasma volume (i.e., FR, see results above), we employed a second normalization strategy using an internal reference miRNA. Normalizing by hsa-miR-103a-3p, we observed a lower variation in the fold abundance of miRNAs between the different isolation protocols studied (S6 Table) than using plasma volume as normalizer (S6 Table and Fig 5). That is, normalization by a endogenous miRNA with similar isolation efficiencies to target miRNA results in similar “fold abundance” values to reference protocol. However, significant differences in miRNAs fold abundance between the isolation protocols used were observed, especially between yQ and yE protocols. Curiously, the highest correlation between protocols was between yQ and yE (r = 0.532, *P*<0.001). As it was mentioned above, these differences are not real because the same twenty plasma samples were used in all isolation protocols. Thus, these differences in fold abundance are apparent fold changes (apFC) and are due to different recovery efficiencies between distinct miRNAs using the same method and furthermore, to different recovery efficiencies for the same miRNA using different isolation methods.

## Discussion

Several groups have compared different isolation protocols and showed that TRizol-based RNA isolation protocols result in highest organic and phenolic contaminants [20]. Furthermore, as previously indicated, these phenol-based isolation protocols result in a poor recovery of structured miRNAs with low GC content from samples with low RNA abundance [28]. Thus, we decided to test a column-based protocol (a modified miRCURY RNA isolation kit Biofluids protocol, Exiqon, E modified protocol), that theoretically is not affected by structural stability and GC content, as well as a combined phenol and column-based protocol (a modified miRNeasy Mini protocol, Qiagen, Q modified protocol), both using the same elution volume.

Furthermore, it is known that the use of RNA carriers improves the miRNA isolation efficiency in plasma samples [26]. We also decided to test two RNA carriers instead of other molecules such as glycogen or linear acrylamide because, although generally these molecules improve Cq values relative to samples without carrier, in some kits they may have no effect (glycogen used with Ambion mirVana kit [22] and linear acrylamide used with the Qiagen miRNeasy kit [25]) or may render higher Cq values (glycogen used with TRIzol isolation followed by column clean-up [22]). Although the use of MS2 RNA as carrier gave the highest total RNA concentration with the Q modified protocol by spectrometry and by electropherogram (S1 and S2 Tables, respectively), the electropherogram analysis showed a smear signal over all RNA regions, which indicated the presence of RNA of a wide range of sizes (S2 Fig). In contrast, the use of yeast RNA as carrier showed a peak in the miRNAs region (S2 Fig) and therefore, showed the highest small RNA and miRNA concentrations, both in Q and E modified protocols (S2 Table). Thus, yeast RNA seems to be more suitable as carrier because it helps to recover mainly the small RNA and miRNA region, improving RNA isolation. On the other hand, the spectrophotometric and electropherogram results of sample isolation in the absence of RNA carrier showed the highest total RNA recovery using the E modified protocol (S1 and S2 Tables). These results were similar to those obtained by Edhl et al [19], using samples with low miRNA abundance and different isolation protocols without carrier.

Since we cannot determine the exact concentration of plasma-derived RNA isolated from samples with a RNA carrier by spectrophotometry or electropherograms, we decided to use an alternative method through estimation of the RNA isolation efficiency. Because UniSp2 was added during the RNA isolation protocol, it was also affected by the isolation protocols and RNA carrier used. Thus, UniSp2 quantification by qRT-PCR was employed as an estimation of the RNA isolation efficiency. We also quantified the UniSp6, which was added during the RT step and was not affected by isolation efficiency. In addition, we quantified different miRNAs with different GC content and miRNA ΔG, all of them affected by isolation protocols and the RNA carrier used. As expected, we did not observe differences in UniSp6 Cq values between different protocols or RNA carriers used. Thus, the differences observed in UniSp2 and several miRNAs studied were due to differences in the isolation protocol, and not because of the presence of inhibitors that may affect the qRT-PCR reaction (Fig 1 and S4 Table).

Despite these data, due to the low A_260/280_ and A_260/230_ ratios obtained by spectrophotometry in our plasma RNA samples (S1 Table), we decided to evaluate the presence of carry-over contaminants derived from the RNA isolation protocol that may affect the qRT-PCR reaction. Firstly, in a serial of tenfold diluted samples, obtained using different isolation protocols and RNA carriers, the UniSp2 Cq values were determined to evaluate the qPCR Eff and thus, the presence of possible qPCR inhibitors. Moreover, we used a UniSp2 standard curve to perform an absolute quantification of all samples, which allowed us to compare their isolation efficiency (S3 Fig and S3 Table). We observed similar UniSp2 qPCR efficiencies between serial tenfold diluted samples and serial tenfold diluted standard UniSp2. These results indicated the absence of qPCR inhibition and hence, we could compare the protocols by isolation efficiencies. We observed the highest UniSp2 recovery and therefore isolation efficiency when a RNA carrier was present (S3 Table), as previously described [26,35]. A combination of phenol and column-based approach (Q protocol) and yeast RNA as carrier exhibited the highest recovery and therefore isolation efficiency, and the lowest coefficient of variation (S3 Table). These results contrast with the results of McAlexander *et al*, who concluded that using glycogen as carrier, Exiqon Biofluids was more efficient than Qiagen miRNeasy Serum/Plasma [22], another biofluids-specific protocol with lower elution volume. These results indicate that each isolation protocol must be optimized with different carriers, as they influence the amount of miRNAs recovered. Finally, regarding the presence of inhibitors in plasma and the inhibition of the qPCR reaction, we must highlight the role of the DNA polymerase used, since different DNA polymerases have distinct tolerance to inhibitors [36]. Consequently, we can not ensure the absence of inhibition of the qRT-PCR reaction using the same miRNA isolation protocols if another qRT-PCR chemistry is used. Therefore, the inclusion of spike-in controls in every step of the procedure and their quantification is crucial to discard the presence of qPCR inhibitors, as we carried out in the present study.

The increase in miRNA isolation efficiency employing RNA carriers can be due to base pair interactions between miRNAs and the RNA carrier. Indeed, we observed the highest recovery of UniSp2 and miRNAs using Q and E modified protocols with RNA carrier (Fig 1 and S4 Table), in accordance with the results reported by Andreasen *et al* [26]. Moreover, in the absence of carrier RNA isolation with the E modified protocol yielded the highest recovery efficiency (Fig 1, S3 and S4 Tables), in agreement with a previous report [24].

We decided to check if the GC content, the free energy of intra-molecular folding and the miRNA SRA influences the recovery of miRNAs isolated using yeast RNA as carrier (i.e., using yQ and yE protocols). For this purpose, we obtained by qRT-PCR the FR of several miRNAs normalized by plasma volume with different GC content and miRNA ΔG (Table 1). We observed significantly higher FR in all miRNAs using yeast RNA as carrier (yQ and yE protocols) compared to without RNA carrier (wQ and wE protocols) (S5 Table). A significant negative correlation was observed between the FR of miRNAs using the yE protocol and the GC content (Fig 2A), and a model of linear regression was performed indicating the contribution of the GC content and the miRNA ΔG. The contribution of miRNA ΔG was clearly visible when the effect of GC content was reduced using miRNAs with GC content between 25% and 75% percentile (Fig 3A), in this case, a slight contribution of the logarithm of miRNA SRA was also observed. On the other hand, a significant positive correlation was observed between the FR of miRNAs using the yQ protocol with the miRNA SRA, and the logarithm of miRNA SRA (Fig 4B). When we removed from the analysis the miRNAs overrepresented in the sample or even when miRNAs with GC content 25% to 75% percentile were used, a negative correlation with the miRNA ΔG was observed, but these correlations were not significant when mean values of FR of miRNAs were used (Fig 3B). This indicates that the use of a RNA carrier increases the recovery of all miRNAs but not in the same proportion, since this recovery depends on the miRNA SRA, the GC content and the miRNA ΔG. It is important to highlight that the contribution of each variable depends on the method employed for RNA isolation. Particularly, the recovery of miRNAs when a RNA carrier is used in yE protocol depends mainly of GC content and miRNA ΔG. However, this recovery in yQ protocol depends mainly of miRNA SRA. These results were coherent with the observation that small RNAs with low GC content and stable secondary structure (i.e., low miRNA ΔG) are isolated less efficiently in samples with low RNA abundance, and that the use of RNA carrier significantly improves their recovery [28]. This is the first study in which the recovery of miRNAs is related to their own relative abundance in the sample and variables such as their GC content and ΔG.

We also analyzed the fold abundance of miRNAs normalized by an internal reference miRNA (hsa-miR-103a-3p). This pattern was different to that observed when miRNAs were normalized by plasma volume (S6 Table). Specifically, we observed that the differences in fold abundance between different protocols are attenuated when we normalized by hsa-miR-103a-3p (S6 Table), in comparison with plasma volume normalization (S5 Table and Fig 5). Even so, significant differences on fold abundance values were observed between protocols. As was mentioned above, these differences in apparent fold changes (apFC) values are due to differences of recovery efficacy of different miRNAs using the same protocol and to differences of recovery efficacy for a miRNA using different isolation protocols. Thus, normalization by an internal reference miRNA showed more consistent results between different protocols than normalization by volume (Fig 5, S5 and S6 Tables). The lowest dispersion in fold abundance of miRNA among isolation protocols observed in our results, coupled with the fact that several authors found significant differences normalizing with internal reference miRNAs but not when we normalized by plasma volume [33,37], lead us to recommend the use of internal reference miRNAs as normalizers.

Thus, the RNA isolation protocol employed and the inclusion of RNA carrier are important analytical variables to take into account, together with other pre- and analytical variables, such as type of sample (serum or plasma) [38–40], anticoagulant used in blood sampling [41], preservation of the sample [38], sample hemolysis [38,42], residual platelet contamination [39], centrifugation procedures [38,39], qRT-PCR chemistry [24,36], or normalization strategies [33,37,43]. All these variables may explain the low inter-laboratory reproducibility [44,45].

In summary, we observed that the use of qRT-PCR is better than the use of absorbance values or electropherogram to compare different RNA isolation protocols. In fact, we can not rule out that results obtained from plasma-derived RNA without carrier on Nanodrop were noise. In the absence of RNA carrier, wE protocol was more efficient than wQ protocol, but in the presence of carrier yQ protocol was more efficient than yE protocol. In our study, yeast RNA carrier renders a more efficient RNA isolationthan MS2 RNA carrier in Q and E modified protocols. Our results point out that the use of yeast RNA as carrier increases the isolation efficiency of miRNAs depending on the relative abundance in the sample, the GC content and ΔG of miRNAs. We also observed that these variables influence differently according to the isolation protocol used. That is, yQ protocol mainly recovered high abundant miRNAs, and yE protocol mainly recovered miRNAs with low GC content and low ΔG and therefore with high intra-molecular thermodynamic stability. In general, we also observed lower variations in fold abundance of miRNAs between protocols when normalizing by an internal reference miRNA instead of normalizing by plasma volume (Fig 5, S5 and S6 Tables). Thus, we recommend normalization of miRNAs levels by a miRNA that previously shows constant Cq values among samples or conditions tested. Finally, a standardization of pre- and analytical conditions is crucial in order to obtain reproducible inter-laboratory results in miRNA studies.

## Supporting information

S1 Fig Optimization of concentration of yeast RNA used as carrier and optimization of input volume used on RT reaction. A) Dilution curve of one plasma-derived RNA sample and isolated with Q modified protocol using different concentrations of yeast RNA carrier (yQ). UniSp2 spike-in was quantified and we observed that from 0.5μg of yeast RNA as carrier did not improve RNA isolation, we also observed that higher carrier amounts did not affect the qPCR reaction. We selected 1μg of yeast RNA as carrier. B) Dilution curve of eight RNA samples derived from plasma and isolated with Q modified protocol with yeast RNA as carrier (yQ) using different input volumes to the RT reaction. UniSp5 spike-in was quantified and we observed that if too much RNA is added to the RT reaction linearity is lost due to the presence of inhibitors of qPCR reaction co-isolated during the sample preparation. We observed that 2μl was the best input volume for the RT-qPCR in our plasma samples.
(PDF)

S2 Fig Electropherogram using the Agilent RNA 6000 Nano Kit for total RNA and the Agilent Small RNA kit for low molecular weight RNA in the Agilent 2100 Bioanalyzer (Agilent Technologies). The same control sample was isolated using different protocols and RNA carriers.
(PDF)

S3 Fig **A) Standard curve from serial dilutions of RT product of UniSp2 of known initial concentration was performed plotting Cq values vs logarithm of UniSp2 concentration (Log Conc).** The equation of the straight line and the correlation coefficient (R^2^) were obtained by linear regression. Using this equation, qPCR efficiency was calculated with the formula: qPCR Eff (%) = 10^(1/slope)^-1 x 100. **Furthermore, we performed serial dilutions of samples (B) to obtain the qPCR efficiency of each protocol. As qPCR efficiencies were similar, Cq values of samples were interpolated to obtain the logarithm of concentration.** Once the UniSp2 sample concentration was known the efficiency of recovery of each protocol and carrier combination was calculated with the formula: Isol. Eff (%) = (UniSp2 CC/UniSp2 TMC) x 100. Being “UniSp2 CC” the absolute concentration calculated by qPCR, and “UniSp2 TMC”, the theoretical maximum concentration (40 pM).
(PDF)

S1 Table Nanodrop RNA concentration (ng/μl), A260/280 and A260/230 absorbance coefficients determined in four RNA samples obtained from plasma after different isolation procedures and with different RNA carriers. (PDF)

S2 Table Electropherogram values from a control sample using the Agilent RNA 6000 Nano Kit for total RNA and for the Agilent Small RNA kit for low molecular weight RNA in the Agilent 2100 Bioanalyzer (Agilent Technologies). The same control sample was isolated using different protocols and RNA carriers.
(PDF)

S3 Table UniSp2 mean concentration recovered (pM), isolation efficiency (Isol. Eff) and qPCR efficiency (qPCR Eff) determined in four RNA plasma samples obtained from plasma after different isolation protocols and with different types of RNA carriers. (PDF)

S4 Table Mean and standard deviation of raw Cq values of the miRNAs analyzed from the samples isolated with the Q and E modified protocols with and without carrier. (PDF)

S5 Table miRNAs values normalized by plasma volume and referred to wE and wQ protocols determined in twenty RNA plasma samples obtained from plasma after different isolation protocols and with different types of RNA carriers. (PDF)

S6 Table miRNAs values normalized by miR-103a-3p and referred to wE and wQ protocols determined in twenty RNA plasma samples obtained from plasma after different isolation protocols and with different types of RNA carriers. (PDF)

## Abbreviations

miRNAs: microRNAs
qRT-PCR: quantitative reverse transcriptase polymerase chain reaction
Isol. Eff: isolation efficiency
qPCR Eff: quantitative PCR efficiency
mE: Exiqon modified protocol using MS2 RNA as carrier
mQ: Qiagen modified protocol using MS2 RNA as carrier
UniSp2: synthetic UniSp2 RNA spike-in
UniSp6: synthetic UniSp6 RNA spike-in
wE: Exiqon modified protocol without carrier
wQ: Qiagen modified protocol without carrier
yE: Exiqon modified protocol using yeast RNA as carrier
yQ: Qiagen modified protocol using yeast RNA as carrier
miRNA ΔG: free energy of the most stable secondary structure of miRNAs
FR: fold recovery of a miRNA taking as reference wQ or wE protocols, in data normalized by plasma volume
apFC: apparent fold change, in data normalized by an endogenous miRNA
miRNA SRA: miRNA sample relative abundance, mean miRNA abundance taking as reference the mean Cq value of all miRNAs used in the wQ or wE protocols
