# Supplementary material for: Comparison of protocols and RNA carriers for plasma miRNA isolation. Unraveling RNA carrier influence on miRNA isolation
Source: PLoS One. 2017 Oct 27;12(10):e0187005. doi: 10.1371/journal.pone.0187005 (PMC5659774; doi:10.1371/journal.pone.0187005)
Supplement: S1 Fig — A) Dilution curve of one plasma-derived RNA sample and isolated with Q modified protocol using different concentrations of yeast RNA carrier (yQ). UniSp2 spike-in was quantified and we observed that from 0.5μg of yeast RNA as carrier did not improve RNA isolation, we also observed that higher carrier amounts did not affect the qPCR reaction. We selected 1μg of yeast RNA as carrier. B) Dilution curve of eight RNA samples derived from plasma and isolated with Q modified protocol with yeast RNA as carrier (yQ) using different input volumes to the RT reaction. UniSp5 spike-in was quantified and we observed that if too much RNA is added to the RT reaction linearity is lost due to the presence of inhibitors of qPCR reaction co-isolated during the sample preparation. We observed that 2μl was the best input volume for the RT-qPCR in our plasma samples. (PDF) [file pone.0187005.s001.pdf]

Supplemental Figures

A) B)

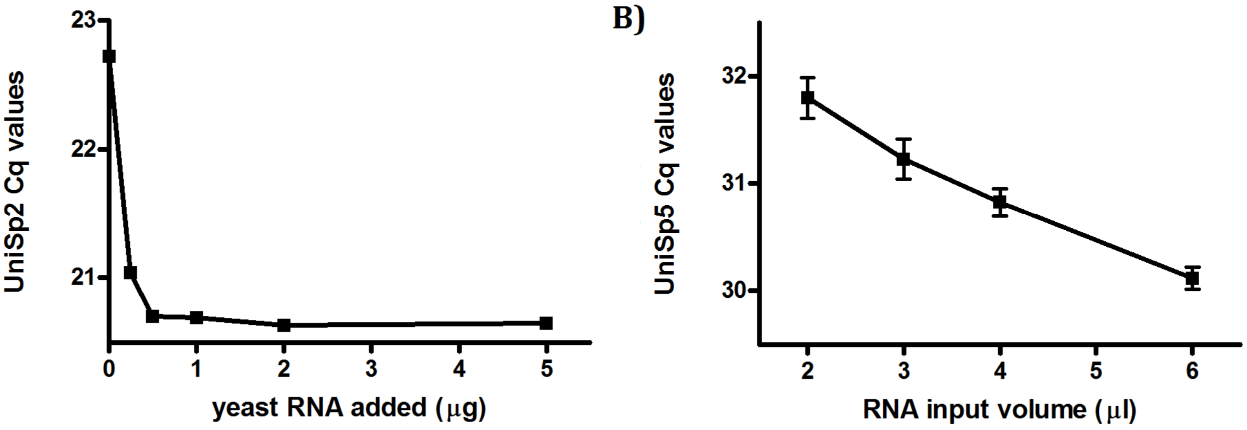

S1 Figure. Optimization of concentration of yeast RNA used as carrier and optimization of input volume used on RT reaction. *A) Dilution curve of one plasma-derived RNA sample and isolated with Q modified protocol using different concentrations of yeast RNA carrier (yQ). UniSp2 spike-in was quantified and we observed that from 0.5µg of yeast RNA as carrier did not improve RNA isolation, we also observed that higher carrier amounts did not affect the qPCR reaction. We selected 1µg of yeast RNA as carrier. B) Dilution curve of eight RNA samples derived from plasma and isolated with Q modified protocol with yeast RNA as carrier (yQ) using different input volumes to the RT reaction. UniSp5 spike-in was quantified and we observed that if too much RNA is added to the RT reaction linearity is lost due to the presence of inhibitors of qPCR reaction co-isolated during the sample preparation. We observed that 2µl was the best input volume for the RT-qPCR in our plasma samples.*
