# Supplementary material for: Comparison of protocols and RNA carriers for plasma miRNA isolation. Unraveling RNA carrier influence on miRNA isolation
Source: PLoS One. 2017 Oct 27;12(10):e0187005. doi: 10.1371/journal.pone.0187005 (PMC5659774; doi:10.1371/journal.pone.0187005)
Supplement: S2 Fig — The same control sample was isolated using different protocols and RNA carriers. (PDF) [file pone.0187005.s002.pdf]

Supplemental Figures

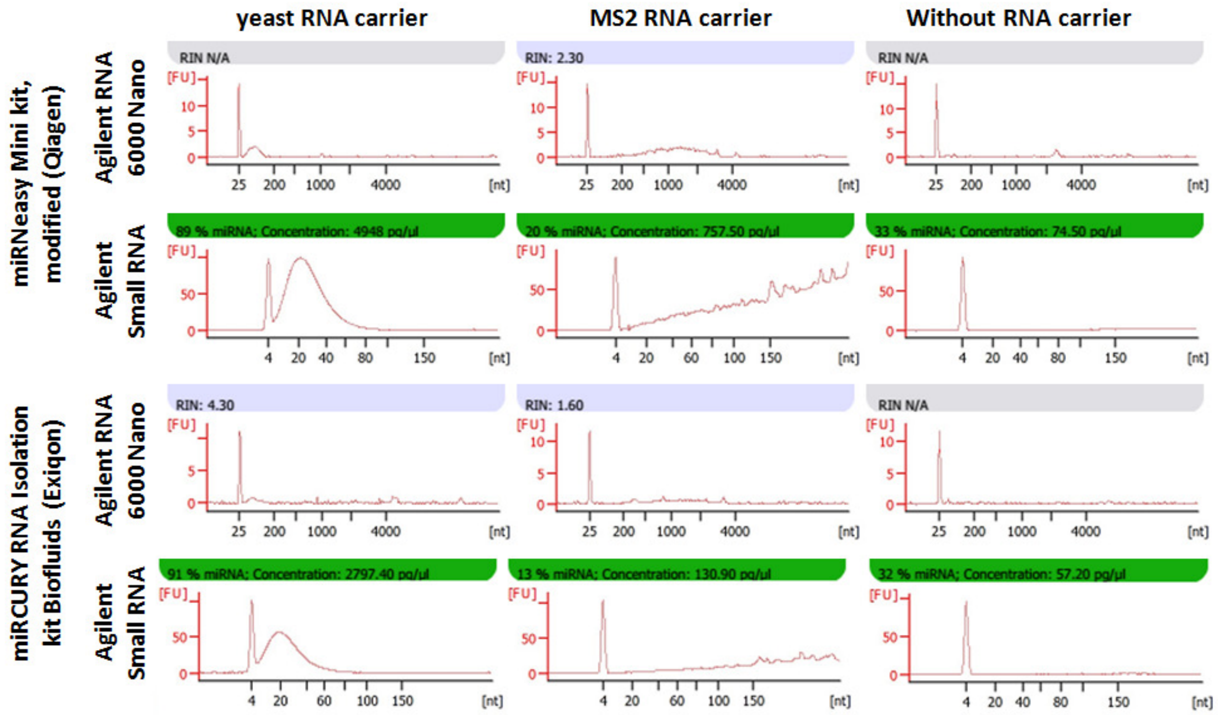

S2 Figure. Electropherogram using the Agilent RNA 6000 Nano Kit for total RNA and the Agilent Small RNA kit for low molecular weight RNA in the Agilent 2100 Bioanalyzer (Agilent Technologies). *The same control sample was isolated using different protocols and RNA carriers.*
