# Supplementary material for: Comparison of protocols and RNA carriers for plasma miRNA isolation. Unraveling RNA carrier influence on miRNA isolation
Source: PLoS One. 2017 Oct 27;12(10):e0187005. doi: 10.1371/journal.pone.0187005 (PMC5659774; doi:10.1371/journal.pone.0187005)
Supplement: S3 Fig — A) Standard curve from serial dilutions of RT product of UniSp2 of known initial concentration was performed plotting Cq values vs logarithm of UniSp2 concentration (Log Conc). The equation of the straight line and the correlation coefficient (R2) were obtained by linear regression. Using this equation, qPCR efficiency was calculated with the formula: qPCR Eff (%) = 10(1/slope)-1 x 100. Furthermore, we performed serial dilutions of samples (B) to obtain the qPCR efficiency of each protocol. As qPCR efficiencies were similar, Cq values of samples were interpolated to obtain the logarithm of concentration. Once the UniSp2 sample concentration was known the efficiency of recovery of each protocol and carrier combination was calculated with the formula: Isol. Eff (%) = (UniSp2 CC/UniSp2 TMC) x 100. Being “UniSp2 CC” the absolute concentration calculated by qPCR, and “UniSp2 TMC”, the theoretical maximum concentration (40 pM). (PDF) [file pone.0187005.s003.pdf]

Supplemental Figures

A)

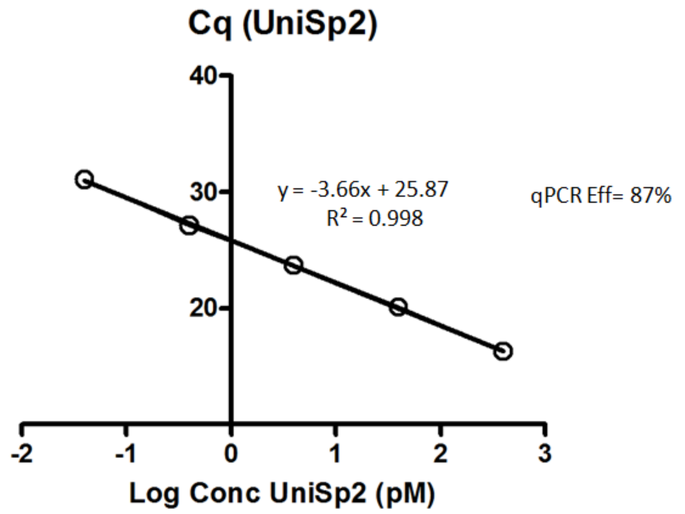

B)

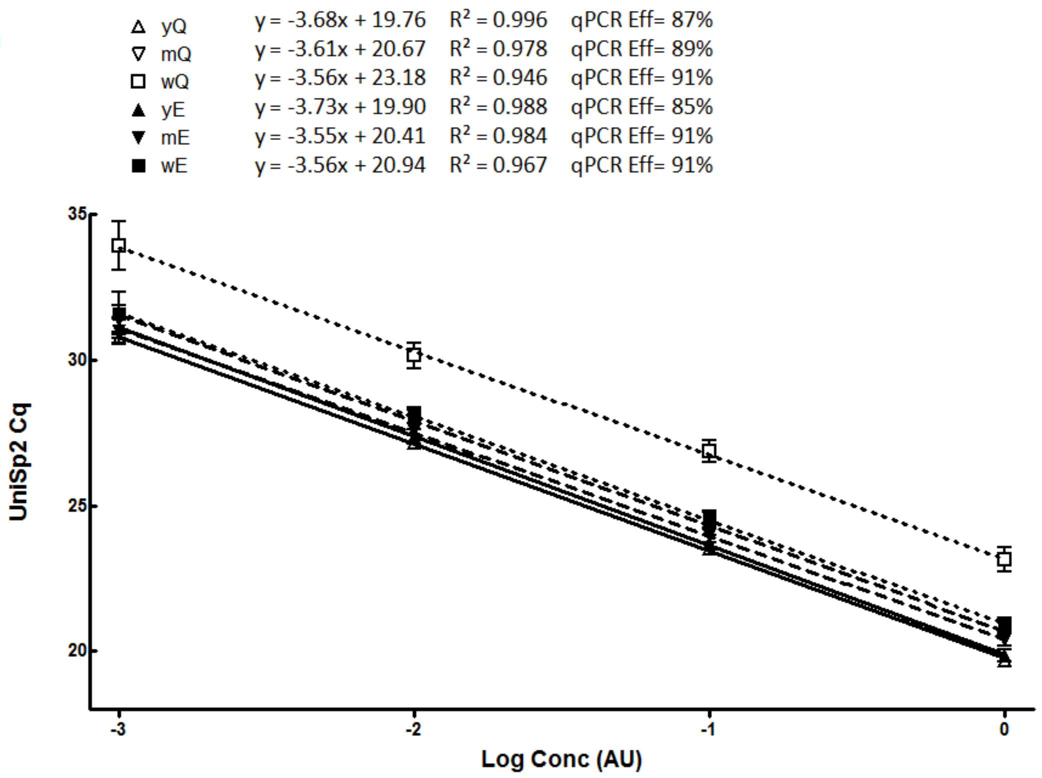

S3 Figure. A) Standard curve from serial dilutions of RT product of UniSp2 of known initial concentration was performed plotting Cq values vs logarithm of UniSp2 concentration (Log Conc). The equation of the straight line and the correlation coefficient (R^2) were obtained by linear regression. Using this equation, qPCR efficiency was calculated with the formula: qPCR Eff (%) = $10^{(1/slope)}$-1 x 100. **Furthermore, we performed serial dilutions of samples B) to obtain the qPCR efficiency of each protocol.** As qPCR efficiencies were similar, Cq values of samples were interpolated to obtain the logarithm of concentration. Once the UniSp2 sample concentration was known the efficiency of recovery of each protocol and carrier combination was calculated with the formula: Isol. Eff (%) = (UniSp2 CC/UniSp2 TMC) x 100. Being “UniSp2 CC” the absolute concentration calculated by qPCR, and “UniSp2 TMC”, the theoretical maximum concentration (40 pM).
