# Supplementary material for: Comparison of protocols and RNA carriers for plasma miRNA isolation. Unraveling RNA carrier influence on miRNA isolation
Source: PLoS One. 2017 Oct 27;12(10):e0187005. doi: 10.1371/journal.pone.0187005 (PMC5659774; doi:10.1371/journal.pone.0187005)
Supplement: S1 Table — (PDF) [file pone.0187005.s004.pdf]

Supplemental Tables

S1 Table. Nanodrop RNA concentration (ng/µl), A260/280 and A260/230 absorbance coefficients determined in four RNA samples obtained from plasma after different isolation procedures and with different RNA carriers.

Protocol		yeast RNA carrier	MS2 RNA carrier	Without carrier
**Q**	**Mean Conc. (ng/µl) ± SD**	14.0 ± 1.5	27.7 ± 5.8	9.4 ± 1.3
	$A_{260/280}$ mean ± SD	1.57 ± 0.10	1.80 ± 0.13	1.31 ± 0.22
	$A_{260/230}$ mean ± SD	0.49 ± 0.12	0.30 ± 0.13	0.17 ± 0.04
**E**	**Mean Conc. (ng/µl) ± SD**	16.3 ± 2.5	24.2 ± 5.7	14.5 ± 9.2
	$A_{260/280}$ mean ± SD	1.29 ± 0.15	1.61 ± 0.09	1.23 ± 0.11
	$A_{260/230}$ mean ± SD	0.17 ± 0.03	0.26 ± 0.05	0.14 ± 0.04

Statistical analysis was done by non parametric Wilcoxon Signed Ranks test with the IBM SPSS Statistics 20 software, non significant differences were obtained between groups analyzed (yQ vs mQ, yQ vs wQ, mQ vs wQ, yE vs mE, yE vs wE, mE vs wE, yQ vs yE, mQ vs mE, and wQ vs wE). y, yeast RNA carrier; m, MS2 RNA carrier; w, without carrier; Q, Qiagen miRNeasy modified protocol; E, Exiqon miRCURY biofluids modified protocol.
