# Supplementary material for: Comparison of protocols and RNA carriers for plasma miRNA isolation. Unraveling RNA carrier influence on miRNA isolation
Source: PLoS One. 2017 Oct 27;12(10):e0187005. doi: 10.1371/journal.pone.0187005 (PMC5659774; doi:10.1371/journal.pone.0187005)
Supplement: S2 Table — The same control sample was isolated using different protocols and RNA carriers. (PDF) [file pone.0187005.s005.pdf]

Supplemental Tables

S2 Table. Electropherogram values from a control sample using the Agilent RNA 6000 Nano Kit for total RNA and for the Agilent Small RNA kit for low molecular weight RNA in the Agilent 2100 Bioanalyzer (Agilent Technologies). The same control sample was isolated using different protocols and RNA carriers.

Protocol	yeast RNA Carrier				MS2 RNA Carrier				Without RNA Carrier			
	total RNA	small RNA	miRNA	ratio	total RNA	small RNA	miRNA	ratio	total RNA	small RNA	miRNA	ratio
**Q**	8	5531	4948	89	31	3832	757	20	6	228	74	33
**E**	11	3089	2797	91	11	1002	131	13	9	181	57	32

Q, Qiagen miRNeasy modified protocol; E, Exiqon miRCURY biofluids modified protocol. Total RNA, total RNA concentration (ng/µl); small RNA, small RNA concentration (pg/µl); miRNA, miRNA concentration (pg/µl); ratio, miRNAs percentage of small RNAs. Total RNA concentration were determined by the Agilent RNA 6000 Nano kit. Small RNA concentration, miRNA concentration and the miRNA/small RNA ratio were determined by the Agilent Small Kit.
