# Supplementary material for: Comparison of protocols and RNA carriers for plasma miRNA isolation. Unraveling RNA carrier influence on miRNA isolation
Source: PLoS One. 2017 Oct 27;12(10):e0187005. doi: 10.1371/journal.pone.0187005 (PMC5659774; doi:10.1371/journal.pone.0187005)
Supplement: S4 Table — (PDF) [file pone.0187005.s007.pdf]

Supplemental Tables

S4 Table. Mean and standard deviation of raw Cq values of the miRNAs analyzed from the samples isolated with the Q and E modified protocols with and without carrier.

Name	yQ (Mean ± SD)	wQ (Mean ± SD)	yE (Mean ± SD)	wE (Mean ± SD)	*P*-value yQ vs wQ	*P*-value yE vs wE	*P*-value yQ vs yE	*P*-value wQ vs wE
**UniSp2**	19,23 ± 0,48	22,55 ± 0,46	19,83 ± 0,48	21,36 ± 0,88	<0,001	<0,001	<0,001	<0,001
**UniSp6**	17,92 ± 0,08	17,93 ± 0,10	17,89 ± 0,11	17,92 ± 0,09	0,735	0,062	0,074	0,991
**let-7a-5p**	30,82 ± 0,59	32,69 ± 1,03	31,16 ± 0,60	32,61 ± 0,97	<0,001	<0,001	0,041	0,869
**let-7b-5p**	31,70 ± 0,75	33,27 ± 0,71	31,26 ± 0,54	31,81 ± 0,77	<0,001	0,012	0,024	<0,001
**let-7g-5p**	31,17 ± 0,98	33,24 ± 1,23	30,66 ± 1,23	32,38 ± 1,58	<0,001	0,002	0,008	0,083
**miR-15a-5p**	29,20 ± 0,76	31,51 ± 0,92	29,10 ± 0,59	30,51 ± 1,08	<0,001	<0,001	0,920	0,002
**miR-16-5p**	24,50 ± 0,78	27,76 ± 1,13	24,69 ± 0,71	26,22 ± 1,36	<0,001	<0,001	0,112	<0,001
**miR-21-5p**	27,57 ± 0,62	29,95 ± 0,79	27,88 ± 0,52	29,64 ± 1,10	<0,001	<0,001	0,068	0,363
**miR-23a-3p**	27,38 ± 0,49	30,33 ± 0,91	27,63 ± 0,73	28,75 ± 0,90	<0,001	<0,001	0,112	<0,001
**miR-23b-3p**	28,66 ± 0,45	31,63 ± 1,02	28,90 ± 0,80	30,07 ± 1,00	<0,001	<0,001	0,273	<0,001
**miR-24-3p**	27,71 ± 0,63	30,68 ± 1,02	28,31 ± 0,63	29,23 ± 0,95	<0,001	<0,001	<0,001	<0,001
**miR-25-3p**	29,17 ± 0,65	31,57 ± 0,89	29,59 ± 0,57	30,50 ± 0,88	<0,001	<0,001	<0,001	<0,001
**miR-30d-5p**	30,55 ± 0,95	32,70 ± 1,01	30,42 ± 0,49	31,56 ± 1,14	<0,001	<0,001	0,920	0,002
**miR-93-5p**	30,79 ± 0,62	33,13 ± 1,06	30,52 ± 0,60	31,48 ± 0,84	<0,001	<0,001	0,028	<0,001
**miR-101-3p**	32,05 ± 1,06	34,14 ± 1,87	31,63 ± 0,70	33,25 ± 1,29	<0,001	<0,001	0,103	0,447
**miR-103a-3p**	29,39 ± 0,41	32,10 ± 0,76	30,07 ± 0,48	31,42 ± 1,06	<0,001	<0,001	<0,001	0,015
**miR-106b-5p**	31,15 ± 0,67	33,73 ± 0,71	31,66 ± 0,71	33,03 ± 1,09	<0,001	<0,001	0,015	0,027
**miR122-5p**	29,55 ± 0,97	31,16 ± 1,08	29,92 ± 1,05	31,09 ± 0,90	<0,001	<0,001	0,006	0,492
**miR-126-3p**	27,77 ± 0,47	30,62 ± 0,69	28,62 ± 0,46	30,10 ± 0,97	<0,001	<0,001	<0,001	0,070
**miR-144-3p**	31,81 ± 0,96	34,25 ± 1,31	31,68 ± 1,17	32,96 ± 1,22	<0,001	0,005	0,404	0,006
**miR-185-5p**	28,73 ± 0,79	31,48 ± 0,85	29,60 ± 0,59	31,01 ± 0,87	<0,001	<0,001	<0,001	0,099
**miR-223-3p**	25,31 ± 0,77	28,08 ± 1,27	25,47 ± 0,71	26,52 ± 0,99	<0,001	<0,001	0,206	<0,001
**miR-320a**	30,22 ± 0,61	31,64 ± 0,63	30,50 ± 0,55	31,62 ± 0,85	<0,001	<0,001	0,068	0,674
**miR-451a**	23,32 ± 0,81	26,81 ± 0,81	23,84 ± 0,62	25,44 ± 0,85	<0,001	<0,001	<0,001	<0,001
**miR-486-5p**	30,78 ± 0,75	32,39 ± 1,13	30,86 ± 0,52	31,57 ± 0,99	<0,001	0,003	0,401	0,002

Statistical analysis was done by non parametric Wilcoxon Signed Ranks test with the IBM SPSS Statistics 20 software. Statistical significan differences are showed in red. y, yeast RNA carrier; m, MS2 RNA carrier; w, without carrier; Q, Qiagen miRNeasy modified protocol; E, Exiqon miRCURY biofluids modified protocol.
