# Supplementary material for: Comparison of protocols and RNA carriers for plasma miRNA isolation. Unraveling RNA carrier influence on miRNA isolation
Source: PLoS One. 2017 Oct 27;12(10):e0187005. doi: 10.1371/journal.pone.0187005 (PMC5659774; doi:10.1371/journal.pone.0187005)
Supplement: S5 Table — (PDF) [file pone.0187005.s008.pdf]

Supplemental Tables

S5 Table. miRNAs values normalized by plasma volume and referred to wE and wQ protocols determined in twenty RNA plasma samples obtained from plasma after different isolation protocols and with different types of RNA carriers.

	**Data referred to wE protocol**								**Data referred to wQ protocol**		
**Name**	**yQ (Mean ± SD)**	**wQ (Mean ± SD)**	**yE (Mean ± SD)**	**wE (Mean ± SD)**	***P*-value yQ vs wQ**	***P*-value yE vs wE**	***P*-value yQ vs yE**	***P*-value wQ vs wE**	**yQ (Mean ± SD)**	**wQ (Mean ± SD)**	***P*-value yQ vs wQ**
**UniSp2**	4,01 ± 1,20	0,39 ± 0,11	2,59 ± 0,91	1,00 ± 0,54	<0,001	<0,001	<0,001	<0,001	10,02 ± 3,05	1,00 ± 0,29	<0,001
**let-7a-5p**	3,24 ± 1,63	0,98 ± 0,90	2,52 ± 1,17	1,00 ± 0,60	<0,001	<0,001	0,019	0,763	3,74 ± 2,44	1,00 ± 0,80	<0,001
**let-7b-5p**	1,08 ± 0,50	0,38 ± 0,22	1,43 ± 0,60	1,00 ± 0,44	<0,001	0,005	0,030	<0,001	2,96 ± 1,56	1,00 ± 0,64	<0,001
**let-7g-5p**	1,92 ± 1,29	0,49 ± 0,34	2,68 ± 1,31	1,00 ± 0,76	<0,001	<0,001	0,011	0,030	4,01 ± 2,80	1,00 ± 0,72	<0,001
**miR-15a-5p**	2,23 ± 1,05	0,48 ± 0,31	2,28 ± 0,79	1,00 ± 0,62	<0,001	<0,001	0,891	<0,001	4,65 ± 2,16	1,00 ± 0,67	<0,001
**miR-16-5p**	2,87 ± 1,47	0,35 ± 0,29	2,45 ± 1,08	1,00 ± 0,62	<0,001	<0,001	0,082	<0,001	8,25 ± 4,13	1,00 ± 0,82	<0,001
**miR-21-5p**	3,65 ± 1,54	0,74 ± 0,38	2,87 ± 1,02	1,00 ± 0,61	<0,001	<0,001	0,030	0,199	4,95 ± 2,01	1,00 ± 0,52	<0,001
**miR-23a-3p**	2,33 ± 0,96	0,34 ± 0,21	2,05 ± 0,90	1,00 ± 0,57	<0,001	<0,001	0,273	<0,001	6,90 ± 2,65	1,00 ± 0,60	<0,001
**miR-23b-3p**	2,32 ± 0,87	0,35 ± 0,22	2,10 ± 0,92	1,00 ± 0,55	<0,001	<0,001	0,312	<0,001	6,68 ± 2,27	1,00 ± 0,61	<0,001
**miR-24-3p**	2,70 ± 1,31	0,39 ± 0,26	1,78 ± 0,92	1,00 ± 0,52	<0,001	<0,001	<0,001	<0,001	6,93 ± 3,03	1,00 ± 0,62	<0,001
**miR-25-3p**	2,39 ± 0,98	0,51 ± 0,40	1,79 ± 0,79	1,00 ± 0,49	<0,001	<0,001	<0,001	<0,001	4,99 ± 2,41	1,00 ± 0,62	<0,001
**miR-30d-5p**	1,97 ± 1,28	0,46 ± 0,33	1,89 ± 0,70	1,00 ± 0,55	<0,001	<0,001	0,812	<0,001	4,27 ± 2,27	1,00 ± 0,62	<0,001
**miR-93-5p**	1,54 ± 0,65	0,37 ± 0,39	1,87 ± 0,88	1,00 ± 0,46	<0,001	<0,001	0,021	<0,001	4,40 ± 1,87	1,00 ± 0,80	<0,001
**miR-101-3p**	2,09 ± 1,02	0,77 ± 0,64	2,93 ± 1,75	1,00 ± 0,63	<0,001	<0,001	0,043	0,492	3,39 ± 2,17	1,00 ± 0,83	<0,001
**miR-103a-3p**	3,35 ± 0,93	0,55 ± 0,25	2,11 ± 0,70	1,00 ± 0,67	<0,001	<0,001	<0,001	0,018	6,08 ± 1,74	1,00 ± 0,45	<0,001
**miR-106b-5p**	3,25 ± 1,46	0,57 ± 0,28	2,39 ± 1,16	1,00 ± 0,62	<0,001	<0,001	0,014	0,014	5,93 ± 2,47	1,00 ± 0,48	<0,001
**miR122-5p**	3,07 ± 2,41	1,05 ± 0,86	2,48 ± 2,15	1,00 ± 0,61	<0,001	<0,001	0,007	0,964	2,92 ± 2,28	1,00 ± 0,82	<0,001
**miR-126-3p**	4,28 ± 1,14	0,63 ± 0,29	2,38 ± 0,70	1,00 ± 0,61	<0,001	<0,001	<0,001	0,039	6,79 ± 1,86	1,00 ± 0,45	<0,001
**miR-144-3p**	2,41 ± 2,02	0,53 ± 0,48	3,11 ± 2,71	1,00 ± 0,60	<0,001	0,002	0,092	0,007	4,41 ± 2,58	1,00 ± 1,05	<0,001
**miR-185-5p**	4,71 ± 2,17	0,75 ± 0,52	2,51 ± 1,15	1,00 ± 0,51	<0,001	<0,001	<0,001	0,186	7,12 ± 4,34	1,00 ± 0,51	<0,001
**miR-223-3p**	2,26 ± 1,42	0,39 ± 0,31	1,98 ± 1,07	1,00 ± 0,56	<0,001	<0,001	0,143	<0,001	5,70 ± 2,92	1,00 ± 0,70	<0,001
**miR-320a**	2,51 ± 0,97	0,95 ± 0,41	2,03 ± 0,70	1,00 ± 0,46	<0,001	<0,001	0,027	0,496	2,65 ± 1,01	1,00 ± 0,44	<0,001
**miR-451a**	4,26 ± 2,13	0,39 ± 0,26	2,83 ± 1,14	1,00 ± 0,54	<0,001	<0,001	<0,001	<0,001	11,46 ± 6,91	1,00 ± 0,55	<0,001
**miR-486-5p**	1,56 ± 0,67	0,62 ± 0,63	1,40 ± 0,47	1,00 ± 0,66	<0,001	0,012	0,172	0,006	2,50 ± 1,06	1,00 ± 0,95	<0,001

Data were referred to wE and wQ protocols. Statistical analysis was done by non parametric Wilcoxon Signed Ranks test with the IBM SPSS Statistics 20 software. Statistical significan differences are showed in red. y, yeast RNA carrier; m, MS2 RNA carrier; w, without carrier; Q, Qiagen miRNeasy modified protocol; E, Exiqon miRCURY biofluids modified protocol.
